# Anatomical Fundamentals and Current Surgical Knowledge of Prostate Anatomy Related to Functional and Oncological Outcomes for Robotic-Assisted Radical Prostatectomy

**DOI:** 10.3389/fsurg.2021.825183

**Published:** 2022-02-22

**Authors:** Benedikt Hoeh, Mike Wenzel, Lukas Hohenhorst, Jens Köllermann, Markus Graefen, Alexander Haese, Derya Tilki, Jochen Walz, Marina Kosiba, Andreas Becker, Severine Banek, Luis A. Kluth, Philipp Mandel, Pierre I. Karakiewicz, Felix K. H. Chun, Felix Preisser

**Affiliations:** ^1^Department of Urology, University Hospital Frankfurt, Goethe University Frankfurt am Main, Frankfurt am Main, Germany; ^2^Cancer Prognostics and Health Outcomes Unit, Division of Urology, University of Montréal Health Center, Montréal, QC, Canada; ^3^Martini-Klinik Prostate Cancer Center, University Hospital Hamburg-Eppendorf, Hamburg, Germany; ^4^Dr. Senckenberg Institute of Pathology, University Hospital Frankfurt, Frankfurt am Main, Germany; ^5^Department of Urology, University Hospital Hamburg-Eppendorf, Hamburg, Germany; ^6^Department of Urology, Koc University Hospital, Istanbul, Turkey; ^7^Department of Urology, Institut Paoli-Calmettes Cancer Centre, Marseille, France

**Keywords:** prostate cancer, anatomy, robotic-assisted, RALP, functional outcome

## Abstract

**Context:**

Meticulous knowledge about the anatomy of the prostate and surrounding tissue represents a crucial and mandatory requirement during radical prostatectomy for reliable oncological and excellent replicable, functional outcomes. Since its introduction two decades ago, robotic-assisted laparoscopic radical prostatectomy (RALP) has evolved to become the predominant surgical approach in many industrialized countries.

**Objective:**

To provide and highlight currently available literature regarding prostate anatomy and to help in improving oncological and functional outcomes in RALP.

**Methods/Evidence Acquiring:**

PubMed database was searched using the following keywords: “robotic-assisted radical prostatectomy,” “anatomy,” “neurovascular bundle,” “nerve,” “periprostatic fascia,” “pelvis,” “sphincter,” “urethra,” “urinary incontinence,” and “erectile dysfunction.” Relevant articles and book chapters were critically reviewed and if eligible, they were included in this review.

**Results:**

New evidence in regards to prostatic anatomy and surgical approaches in RALP has been reported in recent years. Besides detailed anatomical studies investigating the meticulous structure of the fascial structures surrounding the prostate and neurovascular bundle preservation, debate about the optimal RALP approach is still ongoing, inspired by recent publications presenting promising functional outcomes following modifications in surgical approaches.

**Conclusions:**

This review provides a detailed overview of the current knowledge of prostate anatomy, its surrounding tissue, and its influence on key surgical step development for RALP.

## Introduction

Several decades ago, landmark anatomical studies have set the foundations for the current knowledge of the periprostatic anatomy. Since then, several minor and major modifications have been proposed and established, aiming to improve the oncological and functional outcomes of patients who underwent radical prostatectomy. With the advent of robotic-assisted surgery, a more detailed understanding of the prostate anatomy and its surrounding tissue has been achieved owing to the technical magnification and precise robotic instruments (1).

This study is aimed to provide a detailed overview of the current prostate anatomy and its surrounding tissue with its impact on key surgical steps for robotic-assisted laparoscopic radical prostatectomy (RALP).

## Materials and Methods

A search of the PubMed database was conducted to identify literature that addresses the anatomy of the prostate and its adjacent tissues in the context of robotic-assisted radical prostatectomy. No limit was set regarding publication date, however, search emphasis was put on the time period between January 2016 and April 2021, following reviews about general surgical anatomy in prostate cancer patients in 2010 and 2016 published by Walz et al. (1, 2). Potential eligible publications were reviewed, analyzed, and included in the current manuscript after consensus was obtained by all authors.

## Results

### Surgical Approaches for Robotic-Assisted Radical Prostatectomy

To date, several techniques for performing RALP have been proposed in an effort to achieve maximal oncological and functional outcomes. Among those, extraperitoneal and transperitoneal RALPs remain the two most common surgical approaches (3). All approaches aim at minimal damage to the pelvic structures and restoring anatomical and functional relationships in the pelvic floor as closely as possible following radical prostatectomy.

Different approaches to completely preserve anterior retropubic-located structures involved in continence and potency (located in the Retzius space) have been introduced. A transperitoneal Retzius-sparing (RS) RALP approach with posterior access *via* the vesico-rectal pouch was initially proposed by Galfano et al. (4). Small-scaled single-center studies comparing RS and non-RS RALP demonstrated favorable early continence recovery for patients with RS RALP (5). However, ongoing major concerns persist regarding the potential higher rates of positive surgical margins compared to the standard approach, especially in the case of anteriorly located tumors (3, 6). Despite two randomized trials, comparing RS and non-RS RALP, the question of whether RS RALP is associated with a higher rate of positive surgical margins can still not be answered sufficiently (3). Recently, Wagaskar et al. introduced the “hood technique” representing an anterior RALP approach combined with complete preservation of the Retzius-space (7). With this approach, after bladder neck incision, a plane behind the posterior wall of the bladder neck is developed, leaving the Retzius-space untouched. Even though demonstrating outstanding continence rates (83% at 4 weeks after RALP), careful patient selection in regards to tumor location should be performed (7). Of note, further RALP approaches, such as transperitoneal-lateral or transvesical approaches, have been introduced recently and have been precisely analyzed by Martini et al. (3). Additionally, robotic-assisted perineal prostatectomy has been of interest in the recent years demonstrating acceptable functional and oncological outcomes. However, data are derived mainly from single institution small-sized cohorts and should be interpreted with caution (8).

### Anatomy of the Prostate and Adjacent Tissue

**Figure 1:**
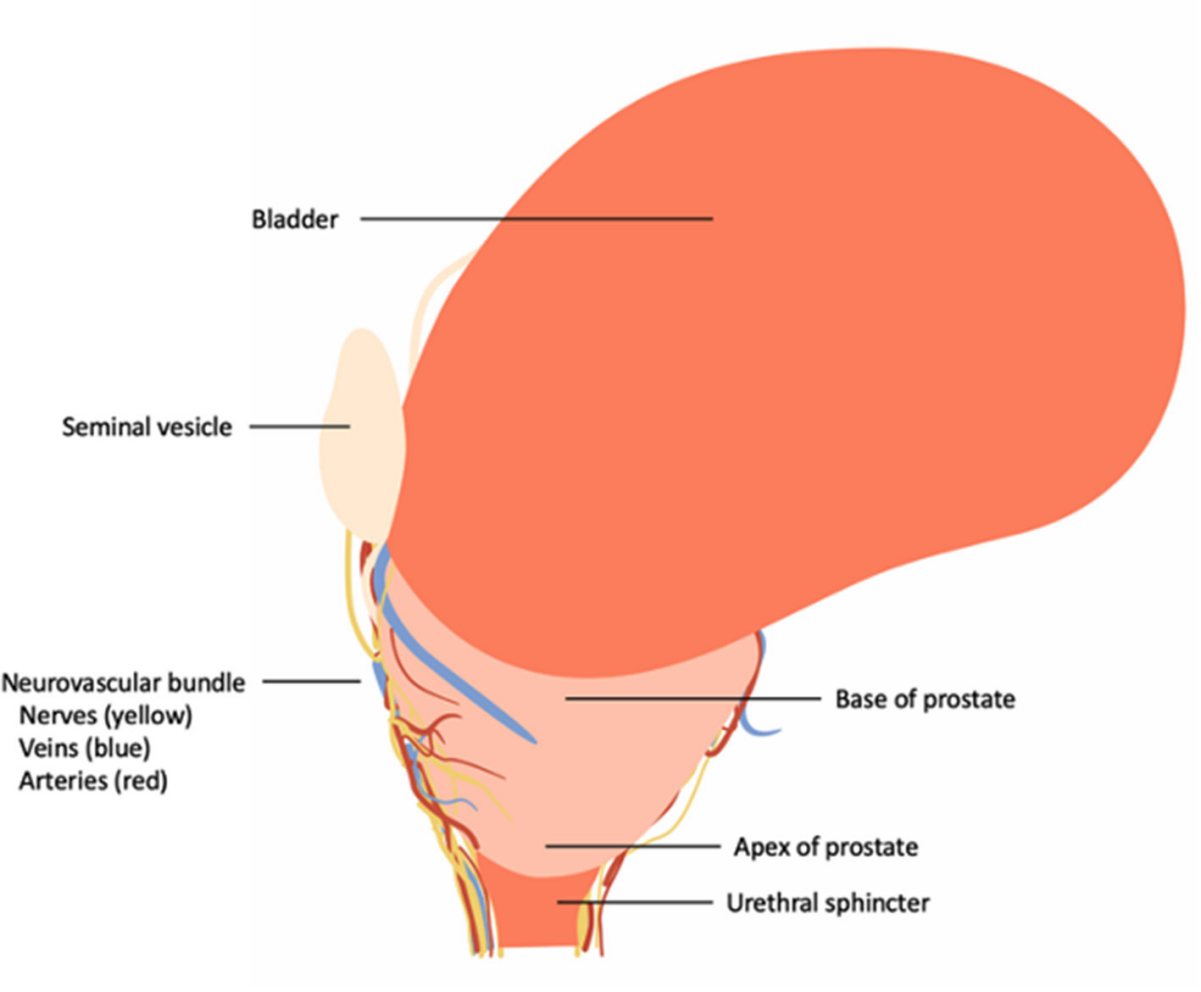
Overview of the topographic location of the prostate and its correlation to the urinary bladder, neurovascular bundle and urinary sphincter complex.

The prostate gland (prostate) is located in the male pelvis and its shape can be considered to be an inverted cone (Figure 1). Its base is in close relation to the bladder neck, whereas the apex is situated in close relationship to the external urethral sphincter (9).

#### Dorsal Vascular Complex

The DVC is located ventrally of the prostate and urethral sphincter containing the dorsal vein complex/*Santorini's plexus* (draining the blood of penile veins) and small arteries, which originate from the inferior vesical artery (10, 11). Ventrally covered by the visceral endopelvic fascia and detrusor apron, DVC may be split at the prostate apex by puboprostatic ligaments (PPL) into a medial and lateral component (12). Ganzer et al. demonstrated in a small case series of human cadaveric studies (*n* = 7) that 37% of the dorsal urethral sphincter at the apex of the prostate and 30% 5 mm distal to the apex are overlapped by the ventral boundaries of the DVC (13). This anatomical knowledge is of utmost importance during DVC ligation, because injury to the urethral sphincter can occur easily, translating into potential postoperative urinary incontinence (1). Data regarding the optimal type of ligation and sequence (delayed vs. *en bloc*) ligation of the DVC are inconsistent and need further investigation. Furthermore, Carvalho et al. reported outstanding functional outcomes (98.4% continence, 86% potency 1-year postsurgery) in a small case series of 128 patients who underwent RALP, relying on a retrograde release of the neurovascular bundle with complete preservation of the DVC and visceral endopelvic fascia (14). Whether these results are based on the surgical technique or due to a study/patient selection bias have controversially been discussed recently, and further, larger-scaled studies are needed to evaluate the optimal surgical DVC approach (14–16).

#### Prostate (Pseudo)-Capsule

The outer “limits” of the prostate are indeed a topic of ongoing controversy (1). The external stromal edge of the parenchyma, formed by fibromuscular layers of condensed transversely arranged smooth muscle, is often labeled as “capsule” (17). From a histological point of view, the correct term for this layer would be “condensed smooth muscle/outer edge.” However, from a surgical point of view, the defined, outer edge of the prostate is visible and grossly apparent in RALP, analogous to a capsule, and can be used as a surgical landmark for precise dissection (1, 18). Walz et al. proposed the term *pseudocapsule* as an acceptable compromise that accounts for both histological and surgical features (1). Contrary to previous findings indicating that the anterior surface of the prostate was absent of a “*capsule*,” Li et al. could demonstrate that the bilateral ends of the capsule were attached to the anterior fibrous muscular stroma/detrusor apron, forming a pocket-like structure for both prostate and the urethra (19).

### Fascial Structures Surrounding the Prostate

#### Parietal and Visceral Endopelvic Fascia

The pelvic organs are covered by a fascia, which can be divided into a parietal and a visceral endopelvic fascia (20). The visceral components of the endopelvic fascia cover the pelvic organs (prostate, bladder, and rectum) and are fused with the anterior fibromuscular stroma of the prostate at the upper ventral aspect of the gland (2, 21). Along the pelvic sidewall at the lateral aspect of the prostate and bladder, the parietal and the visceral components of the endopelvic fascia are fused and are often recognizable as a white-shimmering line called the *fascial tendinous arch of the pelvis*.

#### Periprostatic Fascia

The (visceral endopelvic-derived) fascia on the outer surface of the prostate has been named in different ways throughout previous reports (lateral pelvic fascia, fascia next to the prostate, parapelvic fascia, and prostatic fascia). In line with the most recent reports by Walz et al. the expression “periprostatic” fascia will be used throughout this manuscript. It is noteworthy that the periprostatic fascia cannot be identified as a single-layer stretching over the lateral surface of the prostate. It contains much more in the majority of cases, both collagenous and adipose tissue elements, and depicts as a multi-layered structure (2, 19). The periprostatic fascia may be subdivided into three sections according to the anatomical locations.

#### Anterior Periprostatic Fascia

The anterior element of the periprostatic fascia is located on the anterior surface of the prostate, where it covers the detrusor apron, DVC, and is fused in the midline with the anterior fibromuscular stroma of the prostate (22).

#### Lateral Periprostatic Fascia

The lateral periprostatic fascia usually consists of two separate layers, the laterally located levator ani fascia and an inner fascia covering the pseudocapsule, namely *prostatic fascia*. These layers of fascia, on the anterolateral prostate, extend from the anterior surface of the prostate posteriorly/dorsally to embrace or meet the neurovascular bundle, eventually becoming the pararectal fascia (2, 20, 23). The inner *prostatic fascia* stretches medial to the neurovascular bundle (NVB) to cover the underlying pseudocapsule (21).

The relationship between the abovementioned fascia layers may differ between individuals. Kiyoshima et al. observed that in 52% of cases, no tight adherence between the lateral periprostatic fascia and the pseudocapsule is present (21). The observed space consisted of loose connective and adipose tissue referred to as areolar tissue (24). Li et al. reported in a small case series of human cadaveric studies that on the most convex region of the lateral prostate, both the lateral periprostatic fascia and the pseudocapsule are highly like to fuse into one structure temporarily (19). The authors furthermore highlighted the necessity of performing a careful fascia-pseudocapsule separation in order to the minimize damage to surrounding structures (19).

#### Posterior Prostatic Fascia/Seminal Vesicles Fascia

Both the PPF and SVF cover with a continuous layer the posterior surface of the prostate and the seminal vesicles. These fasciae are also known as “fascia rectoprostatica,” “septum rectovesicale,” “prostatoseminal vescular fascia,” and ubiquitously, *Denonvillier's fascia* (13, 25, 26). Its cephalad origin is found at the caudal end-point of the rectovesical pouch and distends distally to the apex of the prostate at the prostato-urethral junction (2, 27). In line with findings by Muraoka et al. observations by Kim et al. suggest that the tissue quality of PPF/SVF varies among patients as its origin might be induced by tissue tension, created by organ development in the pelvis and not by tissue fusion as suggested previously (1, 28, 29). As this development can vary substantially from patient to patient, the fascia can have a multilayer configuration, fragmentation into short pieces, or be composed of a thick leaf (28). Reconstruction of the posterior musculofascial plate (initially described by Rocco et al. “Rocco stitch”) has been demonstrated to have a beneficial impact predominantly on short-term continence rates (30–33). Whether some specific modifications in the reconstruction approach, such as a 3-layer/2-step approach (including peritoneum) instead of the initially 2-layer/2-step approach by Rocco et al. will result in remarkable continence improvements has to be proven in the future (30, 34).

#### Detrusor Apron and Pubovesical/PPL

Bilateral anterior fibers of the outer longitudinal bladder detrusor reach out over the anterior prostate to the pubis and are referred to as (anterior) “detrusor apron” due to their sheath-type of alignment (1, 25, 26). Recent human cadaveric studies have demonstrated that the detrusor apron splits into three layers of which some contribute to the PPL (35). Together with posterior fibers, the detrusor apron complex helps to attach the urinary bladder in the pelvis but does not actively contribute to the urinary continence mechanism (1, 19, 36). The PV/PPLs are paired fibrous bands inserted on the distal third of the posterior surface of the pubic bone and the anterior bladder and stretch to the urethral sphincter (25). Contrary to the detrusor apron, PV/PPLs, often referred to as PPLs, are considered to play an important part of the suspensory system of continence mechanism (1, 37, 38). Recent findings, derived from human cadaveric studies, indicate that PV/PPL originates both from the visceral endopelvic fascia and the detrusor apron (35). Choi et al. observed in a human cadaveric study (*n* = 31) that PPLs were bilaterally single (61.3%), bilaterally double (19.4%), or mixed (19.4%) prevalent (39). The authors postulate that bilateral double PPLs are likely to result in urogenital competence (39). Due to the close relationship to the DVC and anterior bladder, identification of the PPL is easily appreciated in small/normal-sized prostates. However, it is more challenging to identify it in the presence of concomitant, ventrally expanding benign prostatic hyperplasia (40). Initially introduced by Walsh for open radical prostatectomy (RP) (41), Patel et al. demonstrated that a (anterior) periurethral suspension stitch before DVC dissection was associated with better 3 months continence outcomes compared to no suspension stitch in RALP (*n* = 331) (42). The suspension stitch, secured in the pubic periosteum, was introduced with the aim to maximally preserve the PPF/VSF and stabilize the urethra in its original anatomical position in the pelvic floor. However, it is of note that the statistically significant difference diminished in a longer follow-up time period (42).

### Neurovascular Bundle

#### Inferior Hypogastric Plexus

Nerval structures responsible for the mechanism of erection, ejaculation, and urinary continence originate from the inferior hypogastric plexus (pelvic plexus), which is normally located within a fibro-fatty, sagittal oriented plate between the bladder and the rectum (23, 43–45). Depending on the extend of lymph node dissection (standard vs. extended), surgical intervention is much likely to extend to this area and collateral damage of nerval structures might occur. Differences between standard and extended lymph node dissections relate to the proximal border of dissection. In standard dissection, the common iliac artery or the bifurcation with the ureter proximally is usually considered the proximal border, whereas, in extended approaches, lymph node dissection extends up to the common iliac arteries and to the presacral areas (46, 47). During standard lymph node dissection, inferior hypogastric plexus and erectile nerves are at high risk during dissection in the area of the internal iliac artery toward the bladder region. During extended lymph node dissection, additional risk arises during dissection in the presacral area and medial to the common iliac vessels (48–50).

#### Anterolateral and Posterolateral Nerves of the Neurovascular Bundle

Nerval fibers originating from the inferior hypogastric plexus surround the lateral aspect of the bladder neck, the proximal prostate, and the seminal vesicles (1, 44). Several studies have demonstrated a spray-like nerve distribution during their course on the lateral and anterolateral surface of the prostate (51, 52). Ganzer et al. demonstrated that the largest percentage of periprostatic nerve surface was located in the posterolateral position, and results were later confirmed by Alsaid et al. (53, 54). Clarebrough et al. illustrated that an increased percentage of nerval structures is anterolaterally located at the apex of the prostate compared to the base (11.2 vs. 6.0%) (55). Fibers originating from the posterior parts of the inferior hypogastricus plexus are sometimes referred to as cavernous nerves and can often be found posterolateral to the seminal vesicles (51). These fibers often remain microscopic and are accompanied by vascular structures, resulting in the nomenclature of the *neurovascular bundle* (2). All authors recorded substantial interindividual differences throughout their studies and surgeons should bare potential anatomical aberrance in mind. Even though extended research on the quantity (numbers of nerval fibers) and quality (distribution of parasympathetic nerve fibers) using different methodologies has been conducted previously, the extent of contribution to erectile function is still not sufficiently answered and is part of current research (53, 56).

#### Nerve-Sparing and Grading Systems for Nerve Sparing

**Figure 2:**
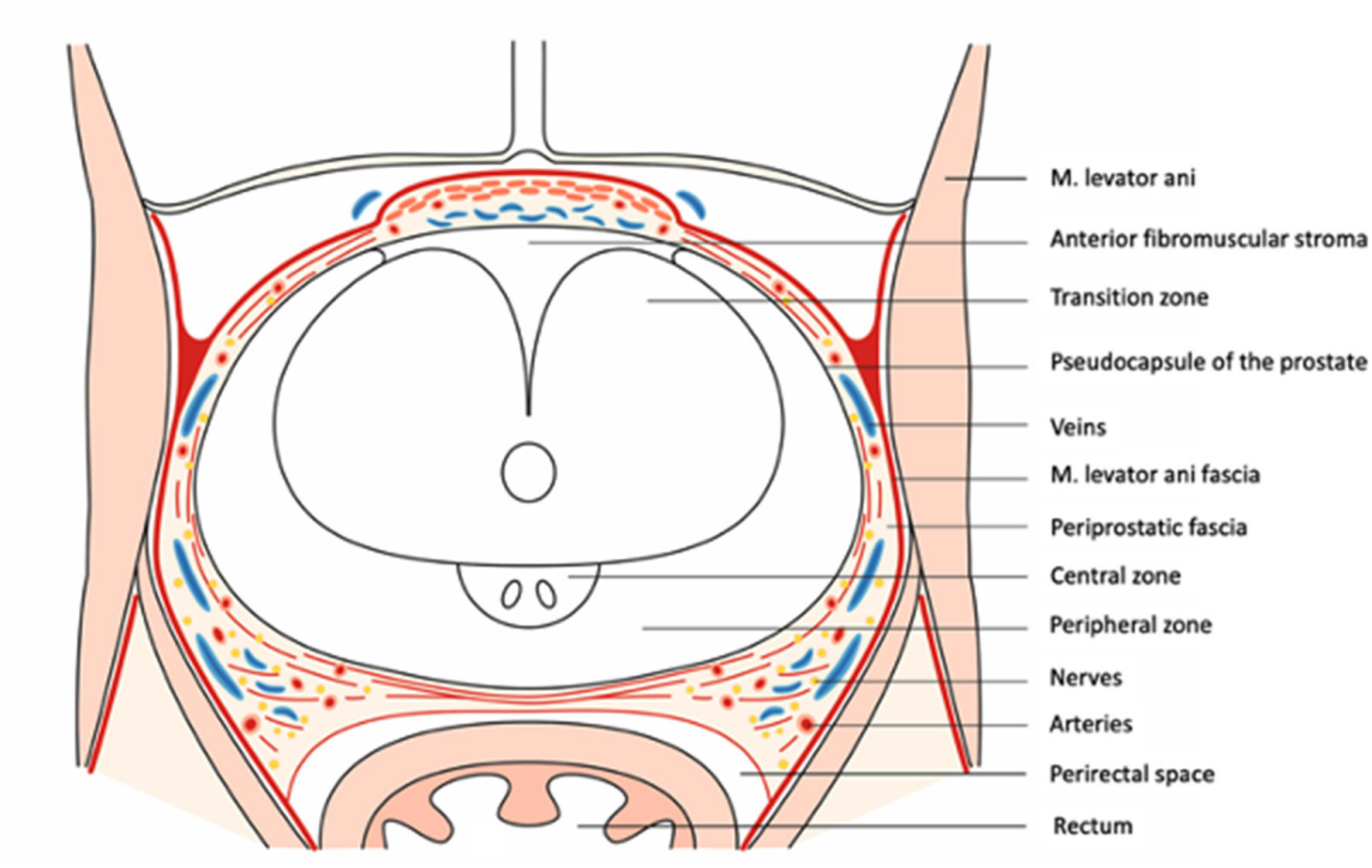
Axial section of the prostate and its adjactent tissue at midprostate.

**Figure 3:**
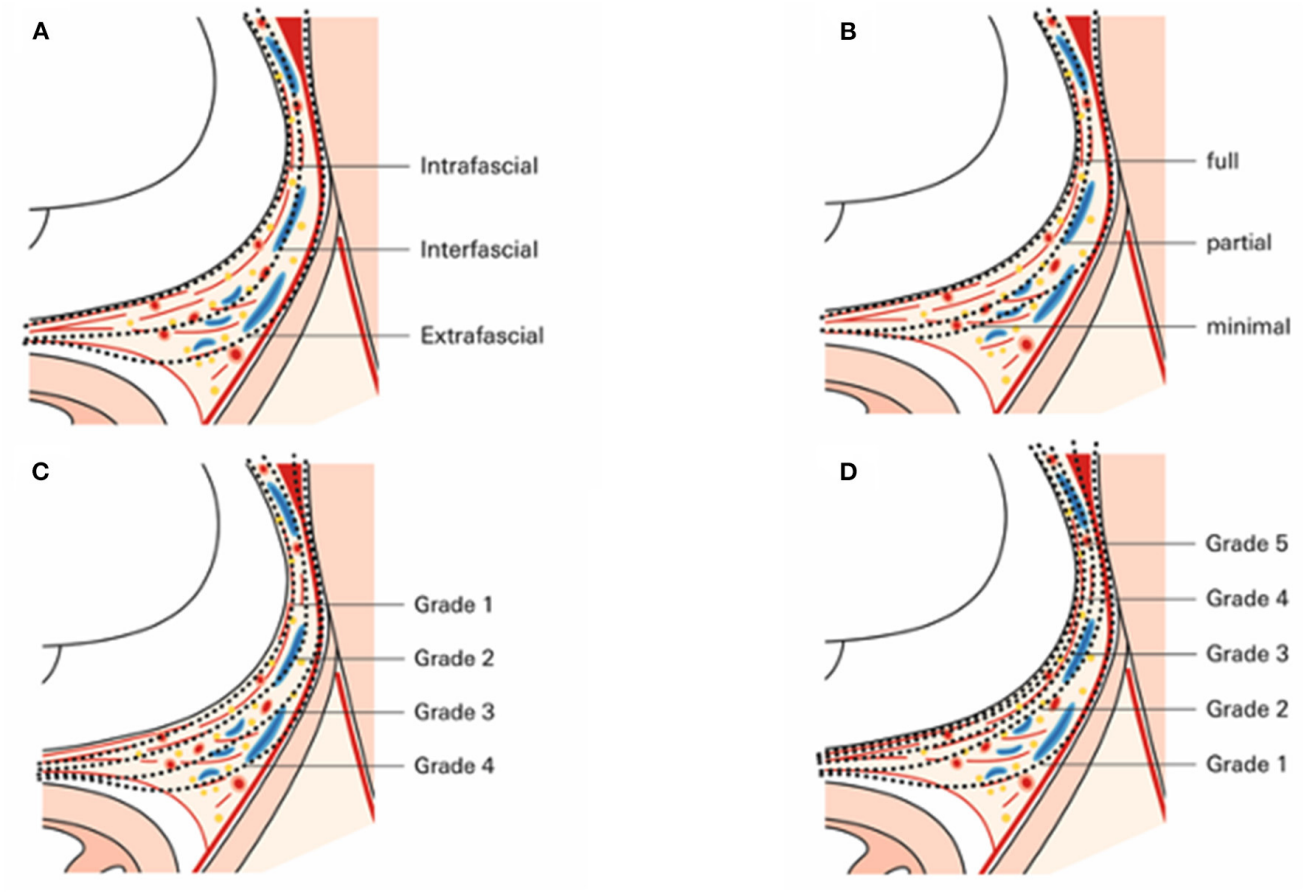
Enlarged axial section of the prostate and its adjactent tissue at midprostate illustrating dissection planes according to **(A)** Walz et al. (1, 2), **(B)** Montorsi et al. (15, 58), **(C)** Tewari et al. (59), and **(D)** Schatthof et al. (60).

Nerve sparing in general aims to preserve a maximum of functional neurovascular tissue that closely surrounds the prostate surface (57). With upcoming knowledge of surgical anatomy and the anatomical relationship between the (peri-) prostatic tissue adjacent, different nerve sparing-grading systems have been established and introduced over the last decade (Figure 2). Walz et al. divided nerve sparing into an intrafascial, interfascial, and extrafascial dissection (Figure 3A), relying on the periprostatic fascia as a guidance and landmark structure while performing nerve sparing (2). An alternative, yet comparable dissection classification was introduced by Montorsi et al. following the *Pasadena Consensus Panel* (58). Herein, three dissections planes (full, partial, and minimal) were suggested (Figure 3B), whereas the minimal dissection plane can be seen as a “sub” extrafascial dissection (1, 58). More recent studies introduced even further differentiation in regards to the dissection planes when nerve sparing is performed. Tewari et al. proposed a grading system based on four grades of dissection (Figure 3C). Relying on both the prostate pseudocapsule and lateral veins on the prostate as surgical landmarks, dissection between periprostatic veins and the pseudocapsule was considered to be grade 1 (highest nerve-sparing quality). By contrast, grade 4 dissection was comparable with an extrafascial dissection (poorest nerve-sparing quality) (59). Besides this four-grade classification system by Tewari et al. and Schatloff et al. proposed an inverse five-grade system for nerve-sparing dissection, in which grade 5 represents optimal nerve sparing and grade 1 represents no nerve sparing (Figure 3D). Contrary to Tewari et al. Schatloff et al. relied on landmark arteries running at the lateral border of the prostate as either prostate or capsular artery. Grade 5 nerve sparing is classified as dissection between this artery and the pseudocapsule without the need of sharp dissection, whereas in grade 4 dissection, sharp dissection in plane between artery and pseudocapsule is necessary (60, 61). Relying on the grading system by Schatloff et al. grade 1 dissection was comparable with an extrafascial dissection (Figure 3D). As an internal validation, Schatloff et al. recorded an inverse correlation between the degree of nerve sparing and the amount of nerve tissue adjacent to the prostate specimen following radical prostatectomy (60).

All grading systems share the concept that the extent of nerve sparing and thus, the functional outcomes have to be weighted to the risk of positive surgical margins, which much likely translates into worse oncological outcomes. Furthermore, incremental nerve sparing with “anatomical landmarks,” such as vascular structures always harbor the risk of intra- and interindividual variability, and thus, applying these nerves sparing grading approaches might not be feasible in all patients to the same extent.

Currently, intraoperative frozen section analyses of the neurovascular tissue-adjacent circumference allow nerve-sparing procedures while simultaneously not comprising oncological outcomes in the vast majority of patients (62, 63). It is of note that besides methodologies relying on intraoperative frozen section, other modalities to predict surgical margin status have emerged recently, such as the usage of confocal laser endomicroscopy to detect positive surgical margins during RALP. Whether these methodologies will represent comparable alternatives to the current methodology needs to be proven in the future (64).

#### Prostate Arterial Supply

The internal iliac artery and its branches supply the pelvis and bifurcate into an anterior and posterior trunk (65, 66). Generally, the anterior trunk gives rise to the superior and inferior vesical arteries, superior and inferior gluteal arteries, and changes into the internal pudendal arteries. Most frequently, prostate arteries rise from the internal pudendal artery (35–56%), followed by the common gluteal-pudendal trunk (15–25%) and branches of the obturator artery or inferior gluteal artery (8–12%) (1, 66, 67). After branching off, the artery supplies several inferior vesical arteries in its course toward the posterior and inferior parts of the bladder, before terminating with numerous prostate branches after a bifurcation, resulting in two main pedicles (1). Different studies have proposed that the anterior pedicle—surrounding the lateral border of the prostate and running to the prostate apex as an anterior capsular prostate branch—may relate to postoperative erectile function and penile integrity (61). It is of note that there is considerable inter- and intraindividual variability in the vascular anatomy of male patients, such as the occurrence of an accessory or aberrant pudendal arteries (4–75%) (68, 69). In a case series with 880 patients who underwent RALP, conducted by Williams et al. transection of accessory pudendal arteries did not turn out to be an independent prognostic factor for postoperative erectile dysfunction (70). Nevertheless, current literature is in agreement that penile blood supply can at least partly originate from accessory pudendal arteries, and thus, attempts to the preservation of these vessels should be performed during radical prostatectomy (70, 71).

### Urinary Sphincter Systems

Two well-recognized urinary sphincter systems play crucial parts in the male voiding mechanism: (a) proximal internal, vesical sphincter, namely, *vesical sphincter* (M. sphincter vesicae) and (b) distal external, urethral sphincter, namely, *urethral sphincter* (M. sphincter urethrae) (1, 2).

#### Bladder Neck and Vesical Sphincter

The anatomical area of the (urinary) bladder outlet into the entrance of the prostatic urethra is referred to as *bladder neck* and is formed by several structures—such as detrusor muscle, vesical sphincter, and adjacent proximal prostatic tissue (1). It is noteworthy that the three-layered detrusor muscles do not participate in forming the vesical sphincter (25). Here, anterior longitudinal muscle fibers reach out over the prostate to the pubis and create the anterior part of the detrusor apron (*Anterior detrusor apron*). Conversely, posterior longitudinal muscles fibers reach out over the bladder neck and insert in the posterior aspect of the prostate (*Posterior detrusor apron*) (1). Both anterior- and posterior detrusor aprons attach the bladder in the pelvis rather than contributing to the sphincteric mechanism (36).

The vesical sphincter, which can be seen as an elliptic structure formed by circular smooth muscle fibers, surrounds the urethral opening circumferentially. However, in general, the opening of the urethra is located eccentrically in the anterior third of the ellipsis, whereas the more posterior located muscles fibers can reach the ureteral orifices (20). While the majority of urinary continence is maintained by the urethral sphincter, a minor component is maintained by the vesical sphincter (72). Nyarangi-Dix et al. demonstrated in a randomized controlled trial that the preservation of the bladder neck resulted in improvements in short- and long-term urinary continence rates (73). These findings were confirmed in a systematic review. However, concerns remain regarding the margin status for prostate cancers located at the prostate base (74).

#### Urethral Sphincter

The urethral sphincter is predominantly located distal to the prostate apex. Irrespectively to the close local relationship to the levator ani muscle, it represents an independent muscle structure and hence, does not relate to the pelvic floor musculature (75). The urethral sphincter consists of two muscle types. Striated muscle fibers at the outer layer, being omega-shaped, extend to the apex, and the anterior of the prostate surface (76–78). Some authors postulated that some parts of this striate musculature stretch not only on the surface of the prostate but also inside the apex of the prostate (78). Additionally, the urethra is completely surrounded by smooth muscle fibers and elastic fibers (75). The proximal extension of these fibers can be located at the colliculus seminalis or verumontanum (78). Following these anatomical observations, a surgical technique, namely, full-length preservation of the urethral sphincter (FFLU technique), was initially reported by Schlomm et al. (78). By identifying and dissecting the striated and smooth muscle part of the urethral sphincter inside the prostate apex until the colliculus, complete preservation of the entire length of the functional urethral sphincter is possible. Relying on this surgical approach, significantly higher rates of continence 1 week following catheter removal (50 vs. 31%) were reported. Interestingly, in the study cohort of Schlomm et al. no differences in long-term follow-up were recorded compared to patients who did not undergo full-length preservation of the urethral sphincter (78).

### Image-Guided Robotic Assisted Laparoscopic Prostatectomy

#### Multi-Wavelength Fluorescence Imaging

Within the last years, novel modalities for imaging guidance during RALP have been studied. The most common application is the use of near-infrared fluorescence (NIRF) imaging during RALP and assists surgeons by identifying vascular anatomy with better accuracy than the naked eye. Relying predominantly on indocyanine green (ICG) as a water-soluble dye, main applications aim to aid neurovascular bundle identification and lymph node dissection (79–81). Future studies will need to clarify the role of fluorescence imaging in the detection of important anatomical structures, especially in the context of sentinel lymph node removal (79).

#### Augmented-Reality in Robotic-Assisted Radical Prostatectomy

Major technological innovations have pathed the way for “precision medicine” in robotic surgery (82). In the context of prostatic surgery, the implementation of AR could increase the understanding of surgical anatomy and facilitate intraoperative navigation during RALP (83). Implementation of results derived from multiparametric resonance imaging of the prostate has already been successfully implemented as real-time AR tools during RALP (83, 84). It is of note that the evolution and improvement of real-time imaging-guided technology are much likely to drastically continue to obtain better oncological and functional outcomes.

## Conclusions

Several notable changes and improvements have been recorded in the last two decades during the advent and establishment of RALP. Among those, a better understanding of the interplay of the periprostatic anatomy and its influence on continence and erectile function have been achieved in the last two decades. Specifically, new insights regarding the relation between periprostatic fascia, urinary sphincteric system, and NVB have been surfaced. This deeper understanding, together with the technical magnification and precise robotic instruments, has led to several surgical modifications and nuances, which were successfully introduced to improve functional and oncological outcomes in RALP. It is speculative but much likely that implementation and broader adoption of enhanced technology, such as intraoperative fluorescence- or AR-guided surgery, will further promote improvements in oncology and functional outcomes in RALP.

Irrespectively of the progress, which has already been achieved in recent years, a statement by Prof. Raychaudhuri and Cahill (85), the pioneer in the development of the anatomic approach to radical prostatectomy, might still nowadays hold true for certain aspects of surgical anatomy for RALP:

“*It is humbling to realize that even today [basic] anatomy may not be known or all understood.”*

## Author Contributions

BH: conceptualization, data acquisition, original draft preparation, and writing–reviewing and editing. LH, MW, and SB: data acquisition. JK and DT: conceptualization. MG: reviewing and editing. AH: supervision and reviewing and editing. JW: conceptualization and data acquisition. MK: writing–reviewing and editing and data acquisition. AB and PM: writing–reviewing and editing. LK: original draft preparation and writing–reviewing and editing. PK: conceptualization and supervision. FC: conceptualization, supervision, and original draft preparation. FP: conceptualization, supervision, and writing–reviewing and editing. All authors substantially contributed to the article and approved the submitted version.

## Conflict of Interest

The authors declare that the research was conducted in the absence of any commercial or financial relationships that could be construed as a potential conflict of interest.

## Publisher's Note

All claims expressed in this article are solely those of the authors and do not necessarily represent those of their affiliated organizations, or those of the publisher, the editors and the reviewers. Any product that may be evaluated in this article, or claim that may be made by its manufacturer, is not guaranteed or endorsed by the publisher.
